# Letter to the Editor

**DOI:** 10.14309/ajg.0000000000004178

**Published:** 2026-09-10

**Authors:** Merel M. Verweij, Michiel T.J. Bak, Annemarie C. de Vries, Ilse Molendijk, Oddeke van Ruler

**Affiliations:** 1 Department of Gastroenterology and Hepatology, Erasmus MC University Medical Center, Rotterdam, The Netherlands;; 2 Department of Surgery, Ijsselland Hospital, Capelle a/d Ijssel, The Netherlands.

We read with great interest the work of Schwartz et al assessing the effectiveness of AVB-114, a bioabsorbable anal plug combined with autologous adipose-derived therapy, in patients with Crohn's perianal fistula (CPF) in the STOMP2 trial (1). Combined remission was defined as closure of the external opening in absence of collections larger than 2 cm on magnetic resonance imaging (MRI). At week 36, this end point was achieved more frequently in patients who received surgical optimization plus AVB-114, compared with those who received surgical optimization alone. Surgical optimization consisted of examination under anesthesia, including fistula curettage and seton placement. Achieving (durable) fistula healing remains a major challenge in this population. As effective treatment options are still limited, these promising findings are of considerable interest (2). However, cautious interpretation is warranted. In this letter, we wish to raise a concern for false positive findings due to the following methodological and reporting issues in this study.

The treatment regimens between the randomized groups differed in important ways. Patients who were treated with AVB-114 underwent a second fistula curettage which may have an effect on the wound healing (3). More importantly, the validity of the Standard of Care (SoC) group as a comparator is uncertain. SoC patients were managed with seton placement alone, and removal depended on the discretion of the treating physician. Because the study does not report on seton-removal rates or timing, the remission rates in this treatment arm are difficult to interpret as patients retaining a seton are, by definition, unable to achieve the primary end point of combined remission.

Second, the sample size calculation was explicitly described as informative only, meaning hypothesis testing was intended to be descriptive rather than confirmatory. Reporting a *P*-value for the primary outcome therefore seems inconsistent with this prespecified aim. The significant difference in remission rates between the AVB-114 and SoC groups at week 36 should therefore be interpreted carefully and may represent a false-positive finding. Furthermore, outcomes of the wait list control cohort who crossed over to AVB-114 treatment after week 36 are not reported, limiting insight into the longer term comparative effectiveness of both strategies.

At last, the terminology of radiologic remission, defined as residual collections of <2 cm on MRI, as it is included in the combined primary end point, is a matter of debate. A more stringent definition for radiologic healing, such as full absence of hyperintensity of T2w-signal, may be more appropriate, given its potential prognostic value and its ability to identify patients with sustained healing (4,5).

To conclude, the work of Schwartz et al highlights AVB-114 as a promising therapeutic strategy for relatively simple CPF. However, given the limitations in study design and the missing information, the conclusion that AVB-114 is superior to SoC should be interpreted with caution. Well-designed and powered trials with stringent end points are warranted to improve outcomes after surgery for patients with CPF and to confirm AVB-114's benefit in broader CPF populations.

## CONFLICTS OF INTEREST

**Guarantor of the article:** Merel M. Verweij, MD.

**Specific author contributions:** M.V.: conceptualization, writing the original draft. M.B., A.C.V., I.M., O.R.: supervision and critical review of the original draft. All authors critically reviewed the literature and approved the final version of the manuscript.

**Financial support:** None to report.

**Potential competing interests:** Michiel T.J. Bak has served as speaker for Abbvie and received an unrestricted research grant from Falk Pharma BeNeLux, outside the submitted work. Ilse Molendijk served on advisory boards for and received financial support from Abbvie, Dr. Falk and Janssen-Cilag outside of the submitted work. Annemarie C. de Vries served on advisory boards for Takeda, Janssen, Bristol Myers Squibb, Abbvie, Pfizer, Lilly and Galapagos and received unrestricted research grants from Takeda, Janssen, Lilly and Pfizer, outside the submitted work. Oddeke van Ruler has served as invited speaker for Janssen-Cilag, has received financial support from Janssen-Cilag and Takeda, and serves as a proctor for Arthrex outside the submitted work.
